# Undiagnosed Subacute Bioprosthetic Aortic Valve Abscess Leading to Complete Heart Block Requiring Permanent Pacemaker Insertion: A Case Report

**DOI:** 10.7759/cureus.87653

**Published:** 2025-07-10

**Authors:** Kamran Dawood, Faraz S Rana, Zahid Khan, Arun Ranjit

**Affiliations:** 1 Cardiology, Mid and South Essex National Health Service (NHS) Foundation Trust, Southend-on-Sea, GBR; 2 Cardiology, Aintree University Hospital, Liverpool University Hospitals National Health Service (NHS) Foundation Trust, Liverpool, GBR; 3 Cardiology, University of South Wales, Pontypridd, GBR; 4 Cardiology, University of Buckingham, London, GBR; 5 Cardiology, Barts Heart Centre, London, GBR

**Keywords:** antibiotic administration in the management of severe sepsis, device-associated endocarditis, minimally invasive aortic valve replacement, organ failure from sepsis, permanent pacemaker implantation (ppm), s. anginosus endocarditis, surgical aortic valve replacement (savr), trans aortic valve replacement, transcutaneous aortic valve replacement, transthoracic echocardiogram

## Abstract

Aortic root abscess is a rare and serious complication of infective endocarditis (IE). The presentation can be vague, and a high degree of suspicion is usually required. We present the case of a 78-year-old woman with a previous history of aortic valve replacement (AVR) surgery eight months ago who presented to the Accident and Emergency (A&E) department after falling at home. After initial scrutiny, her electrocardiogram (ECG) showed complete heart block, for which she received a dual-chamber permanent pacemaker. At that time, due to suspected chest infection, her laboratory tests showed slightly elevated C-reactive protein levels, but blood cultures and chest radiography did not show any evidence of infection. She was administered oral antibiotics for a chest infection and underwent successful pacemaker implantation. After approximately a week, she presented again with generalized weakness and collapse without loss of consciousness. Repeated blood tests showed elevated inflammatory markers, and blood cultures were positive for *Streptococcus anginosus*; therefore, intravenous antibiotics were administered*. *Transesophageal echocardiography (TEE) revealed an aortic root abscess, and ECG revealed a normal sinus rhythm. She also underwent pacemaker interrogation, which revealed normal pacemaker function. She also had a recent overseas travel history and required intensive care admission for a chest infection approximately two months ago, and she had been feeling generally unwell since then. The patient underwent tissue AVR (TAVR) for degenerative calcific aortic stenosis (AS) without periprocedural complications and recovered well after surgery. She was discussed in a multidisciplinary team meeting and was referred for early redo AVR. Unfortunately, the patient died during admission due to sepsis prior to undergoing redo valve surgery.

## Introduction

Aortic root abscess is one of the most serious complications of aortic valve endocarditis, requiring early surgery, which improves long-term survival [1]. The key risk factors for the development of infective endocarditis (IE) include a history of IE, prosthetic devices/implants/valves, immunocompromised states, and congenital heart disease. An aortic root abscess may cause persistent sepsis, heart failure, conduction abnormalities, fistula formation, and an increased risk of embolic phenomena, leading to increased mortality and morbidity in these patients [2]. Clinical history, examination, and laboratory tests, including blood cultures and serological testing, were used to diagnose IE cases. Over the last several decades, the modified Duke criteria and echocardiography have remained the cornerstone for supporting and confidently establishing a diagnosis of IE [3,4]. The modified Duke criteria consist of major and minor criteria. The major criteria include positive blood cultures for typical microorganisms responsible for IE, microorganisms consistent with IE from persistently positive blood cultures for typical or atypical organisms, and echocardiographic evidence of IE. The minor criteria include fever of temperature > 38°C; vascular phenomena such as Janeway lesions, arterial emboli, mycotic aneurysm, intracranial hemorrhage, and septic pulmonary infarcts; immunological phenomena (e.g., Roth's spots, Osler's nodes, glomerulonephritis, and rheumatoid factor); microbiological evidence; and predisposing heart condition or intravenous (IV) drug use. We report a unique case of subacute bacterial prosthetic valve endocarditis (PVE) that presented with a fall and complete heart block. She was treated with a permanent pacemaker (PPM) in the context of a complete heart block. After a few days, she was readmitted with generalized malaise, temperature spikes, and falls. During the workup, she was noted to have an aortic root abscess, and blood culture revealed *Streptococcus anginosus* for which she received IV antibiotics and was referred for repeat aortic valve replacement (AVR) surgery.

## Case presentation

**Figure 1:**
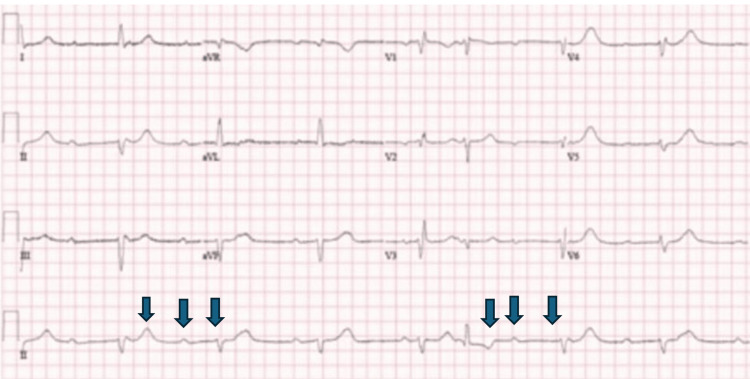
Electrocardiogram showing complete heart block with dissociation between p-waves and QRS complexes as shown by arrows

A 78-year-old woman presented to the Accident and Emergency (A&E) department after falling at home. She complained of feeling dizzy, with her legs giving way at home without experiencing loss of consciousness. The patient was hemodynamically stable at the time of presentation, and her physical examination was unremarkable. An electrocardiogram (ECG) revealed a complete heart block (Figure 1). Laboratory tests showed an elevated C-reactive protein (CRP) level of 57 mg/L (reference: <4 mg/L). Her chest radiograph was normal, and the blood cultures were negative. She had a mild cough and was treated empirically with oral antibiotics for a possible lower respiratory tract infection in the absence of any obvious source of infection. The patient underwent successful PPM implantation during admission and was subsequently discharged.

Her medical history was significant for a bioprosthetic AVR, which was implanted eight months ago, secondary to severe calcific degenerative aortic stenosis. Notably, she had recently travelled to a remote island two months prior. She had a respiratory tract infection and was admitted to the intensive care unit for two days, followed by a step-down to the respiratory ward, and was treated with IV antibiotics. She continued to experience malaise and fatigue after discharge but remained afebrile. After returning to the United Kingdom, she visited her general practitioner (GP), who prescribed a further course of oral antibiotics, but to no avail. The patient did not have an echocardiogram or computed tomography (CT) scan at this time to look for the possible source of infection.

Approximately three weeks after receiving PPM, she was admitted to the hospital with complaints of fatigue, malaise, and an episode of fall at home, without loss of consciousness. Physical examination revealed a (new) soft early diastolic murmur in the aortic (A2) area that was not heard or documented during previous hospital admission. The patient was hemodynamically stable, with no signs or symptoms of heart failure. Pacemaker interrogation revealed normal device function, and the ECG showed a paced rhythm. Her CRP level was high (190 mg/L) with normal white blood cell and neutrophil counts. Blood cultures were positive for *Streptococcus milleri*, a subgroup of the *S. anginosus* group of bacteria. She was started on IV piperacillin-tazobactam three times daily after discussion with a microbiologist and later switched to IV benzylpenicillin 1.2 g six times daily.

**Figure 2:**
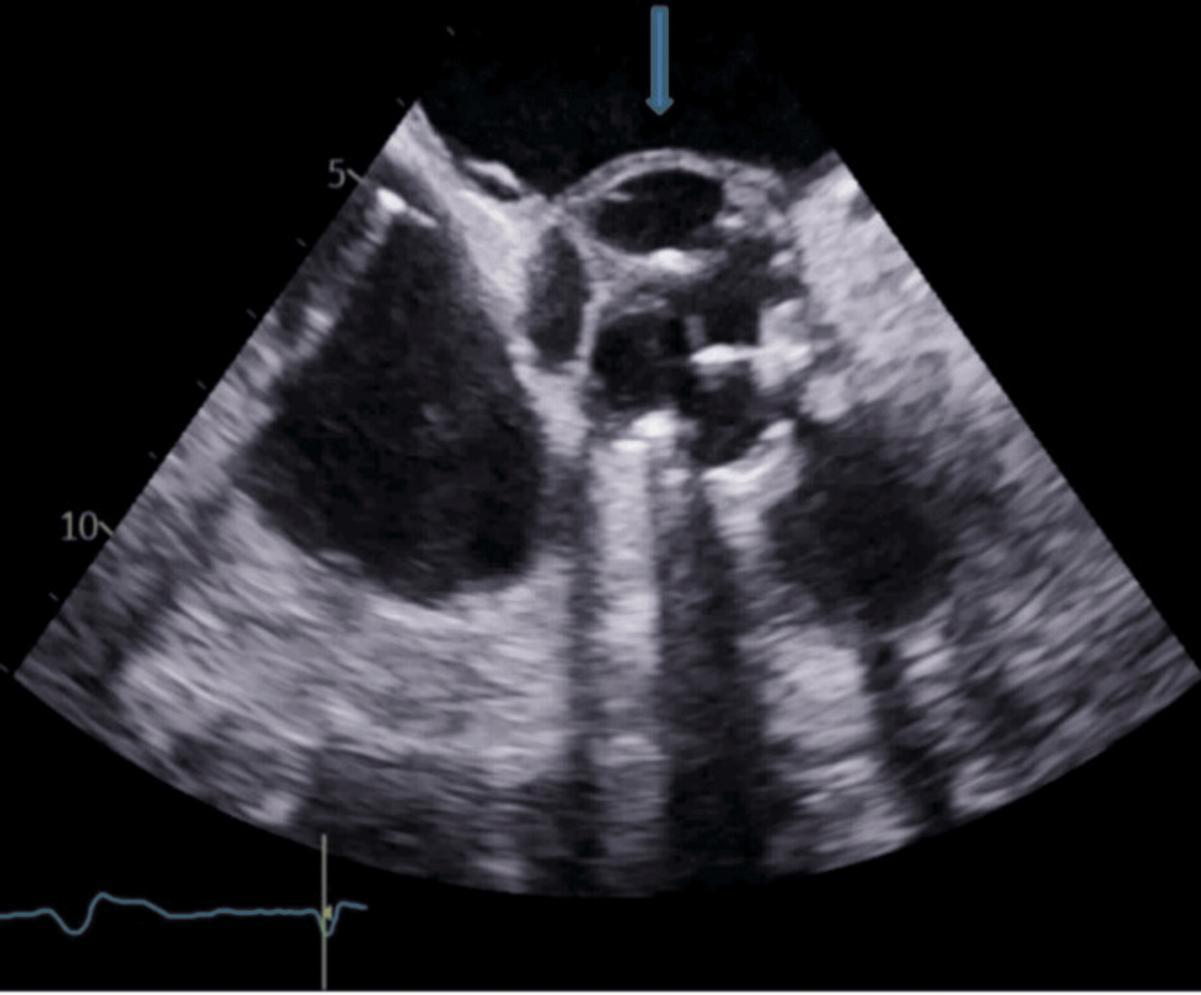
Transesophageal echocardiogram showing aortic root abscess (pointed arrow)

**Figure 3:**
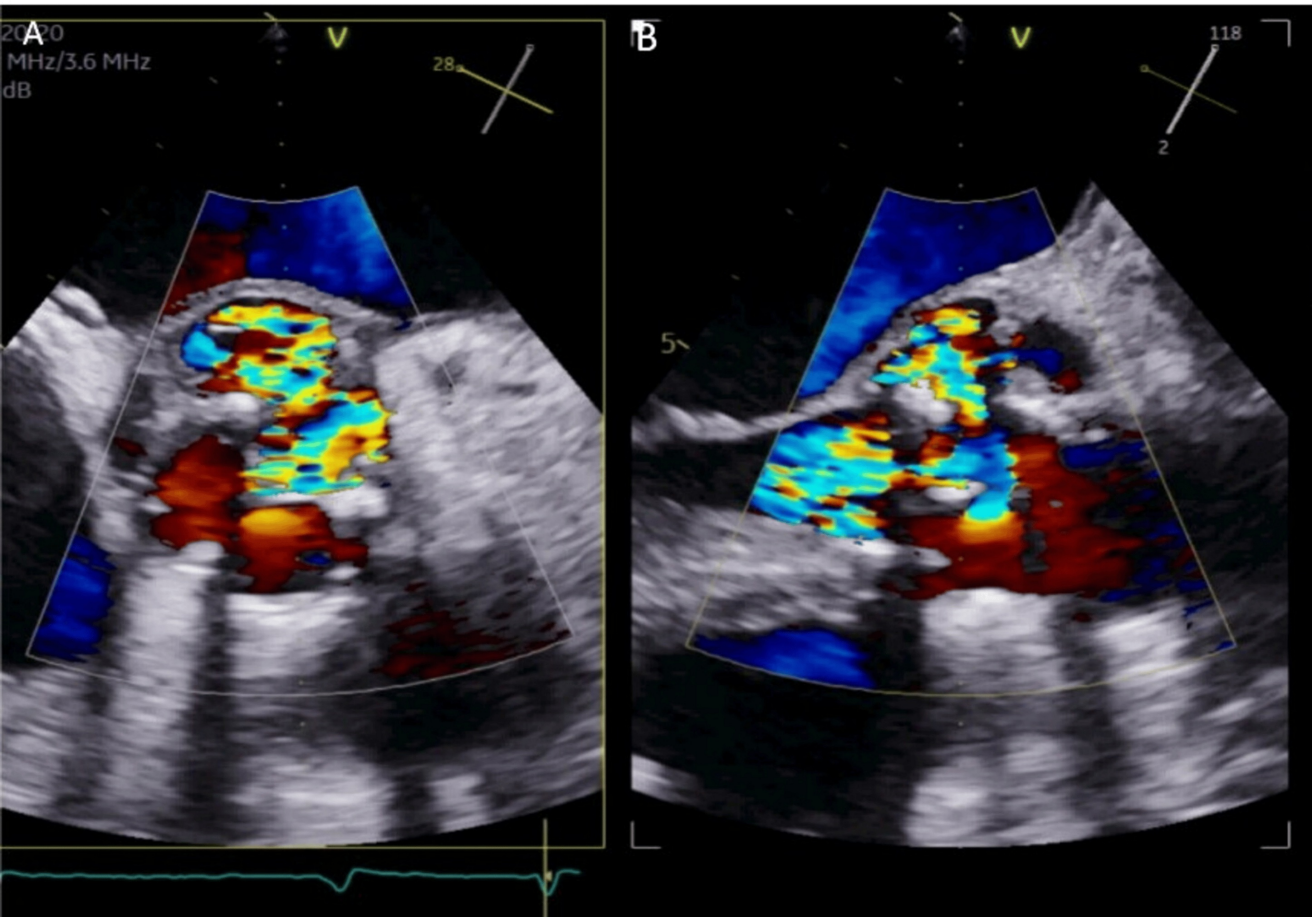
Transesophageal echocardiography showing moderate-to-severe aortic regurgitation

Transthoracic echocardiography revealed a well-seated bioprosthetic aortic valve with moderate-to-severe aortic regurgitation (AR) and echo-free space around the annulus. Her case was discussed at an IE multidisciplinary team (MDT) meeting, and she underwent transesophageal echocardiography (TEE). TEE also revealed an aortic root abscess with moderate-to-severe AR (Figures 2, 3). She was then referred to the cardiothoracic surgical team for early aortic valve debridement and redo AVR. The patient remained quite unwell, and she was rediscussed in the MDT, which recommended optimal medical management owing to the guarded prognosis in this case. The patient continued to receive IV antibiotics; however, unfortunately, in this case, the patient did not survive severe sepsis caused by IE and died.

## Discussion

Aortic root abscesses are one of the most serious complications of IE. Contemporary data suggest that of all the valves, perivalvular abscesses are more commonly seen in aortic valve IE (10%-40% in native valve endocarditis (NVE)) [5-7] and are a frequent finding in PVE (56%-100%) [5]. The in-hospital mortality rate for patients with PVE may be as high as 20% for surgically implanted valves [8]. Glaser et al. used the SWEDEHEART (Swedish Web-System for Enhancement and Development of Evidence-Based Care in Heart Disease Evaluated According to Recommended Therapies) register to examine data from 26,850 patients and found that the prevalence of bioprosthetic PVE was higher than that of mechanical PVE (8.6% vs. 7.3%) [9]. This is also supported by a large American observational study of >38,000 patients (2.2% vs. 1.4%) [10].

The main risk factors for PVE include previous NVE, type of prosthetic valve implanted, prolonged cardiopulmonary bypass time, and male sex [10-12]. Some of the modifiable infectious sources in post-operative patients include intravascular catheter infection, urinary tract infection, chest infections, and sternal wound infections. These patients may present with vague symptoms such as fever, new-onset cardiac murmurs, chills and rigors, and thromboembolism [10,12]. The risk factors for transcutaneous AVR include high body mass index, diabetes mellitus, liver cirrhosis, pulmonary disease, peripheral artery disease, and chronic kidney disease [10].

A case series of two patients reported that the modified Duke criteria may not be sufficient for diagnosing tissue AVR (TAVR)-IE, and TEE may be negative or indeterminate in patients with TAVR-IE, as prosthetic valve shadows may obscure smaller vegetation or abscesses. Positron emission tomography (PET) and CT may be helpful in these patients, and multimodal imaging should be considered in addition to the modified Duke criteria to facilitate early diagnosis and management [13].

TEE provides useful anatomical definitions, such as the extent of annular involvement and extension of the abscess to the subaortic curtain or upper interventricular septum. These are essential considerations when planning surgery in these patients. If an aortic root abscess is detected, urgent surgery is required to prevent further complications. Aggressive debridement of all infected and devitalized tissues is the mainstay of surgical treatment [14]. Reconstruction of the left ventricular outflow tract with autologous pericardium or aortic valve translocation may be required in some cases.

This patient, who had never felt completely well since she acquired a chest infection, clearly depicted a case of subacute bacterial endocarditis (complicated to aortic root abscess) secondary to it. Her diagnosis was delayed because she developed a positive blood culture for a bacterium that commonly involves the mucous membranes of the oropharynx and the respiratory tract, and she did not have an echocardiogram on her first admission when she was admitted with complete heart block. Patients with prosthetic heart valves and raised inflammatory markers should have further screening to look for IE and aortic root abscess, as these patients may not present with typical IE features. The complete heart block presentation in her case was more likely due to IE rather than the more common cause of complete heart block due to iatrogenic damage to the adjacent conduction system tissue. The persistent underlying “subacute abscess” was the likely cause for her complete heart block, which was missed at the time of her pacemaker implantation, and an echocardiography prior to pacemaker implantation would have been helpful in her case.

## Conclusions

Our case highlights the importance of considering subacute IE as an important differential diagnosis in patients presenting with non-specific symptoms and a history of AVR. Even if the initial blood cultures are negative, the suspicion of subacute IE should remain high. More specific take-home messages in the context of the right clinical setting include subacute bacterial IE in patients who are unwell and present with complete heart block, and the importance of early imaging, particularly TEE, to rule out aortic root abscesses in such patients. Early surgical intervention is vital in patients with subacute left-sided NVE or PVE with an aortic root abscess.
